# Effects of HuoxueJiangtang decoction alone or in combination with metformin on renal function and renal cortical mRNA expression in diabetic nephropathy rats

**DOI:** 10.1080/13880209.2020.1844242

**Published:** 2020-11-15

**Authors:** Xuemei Liu, Deliang Liu, Youyou Shuai, Huilin Li, Hengxia Zhao, Xin Qu, Shufang Chu, Xuewen Zhang

**Affiliations:** a Department of Endocrinology, Shenzhen Traditional Chinese Medicine Hospital, Shenzhen, Guangdong, China; b Institute of National Master of TCM, Shaanxi University of Chinese Medicine, Xianyang, Shaanxi, China

**Keywords:** Blood glucose, biochemical indicator, pathological change, kidney

## Abstract

**Context:**

HuoxueJiangtang decoction (ZY) is a traditional Chinese medicine for the treatment of diabetes.

**Objective:**

The protective effect of ZY on renal injury in diabetic nephropathy rats was investigated in this study.

**Materials and methods:**

Fifty 4-week-old SPF Wistar male rats were selected to construct diabetic nephropathy model rats (DN) group by continuous high-fat feeding for 4 weeks, followed by a tail vein injection of 30 mg/kg streptozotocin for 1 week. The experimental rats were divided into six groups of 10 rats: normal (control), DN, DN + ZY, DN + metformin, DN + metformin + ZY, and DN + metformin + captopril (positive control) groups. Among the groups, 6.25 g/kg ZY, 250 mg/kg metformin, and 17.5 mg/kg captopril were given to the rats by gavage once a day for 16 weeks. Blood glucose, dietary behaviour, biochemical indicators, and gene expression changes were measured in each group.

**Results:**

Metformin + ZY treatment significantly lowered blood glucose, water intake, urine total protein, urine albumin, urine volume, serum triglyceride, and serum cholesterol levels in the DN group. The pathological changes of kidney tissue showed that the DN + metformin + ZY group had a protective effect on kidney tissue damage. And ZY and metformin + ZY treatments repaired the expression of genes in the DN group.

**Discussion and conclusion:**

The ZY and metformin combined treatment showed a clear therapeutic effect on kidney damage in DN. This study provides a potential mechanism for the treatment of diabetic nephropathy with ZY combined with metformin.

## Introduction

Diabetic nephropathy affects approximately 9% of adults worldwide and is a common complication of type 1 and type 2 diabetes. The clinical features are proteinuria, hypertension, and impaired renal function (World Health Organization News 2014). Diabetic nephropathy is also common in patients with type 2 diabetes mellitus, which is the main cause of renal replacement therapy due to renal failure worldwide and also the most common cause of death in diabetic patients.

The treatment of patients with diabetic nephropathy is divided into four main areas: blood glucose control with oral hypoglycaemic agents or insulin, and diet control; reducing cardiovascular risk by smoking cessation and lipid-lowering therapy; blood pressure control using angiotensin receptor blocker therapy; and inhibition of renin–angiotensin system (RAS) treatment strategies by the use of RAS inhibitors, angiotensin-converting enzyme inhibitors, renin inhibitors, and mineralocorticoid antagonists, which have demonstrated beneficial effects on diabetic nephropathy in animal and clinical trials (Umanath and Lewis 2018).

Traditional Chinese medicine has also been found to have a therapeutic effect on diabetic nephropathy. For example, yacon leaf decoction has been suggested to exert beneficial effects on diabetic nephropathy rats (Honore et al. 2012). In addition, LiuweiDihuang decoction reverses diabetic nephropathy symptoms such as decreased blood glucose and impaired renal function (He et al. 2007; Honore et al. 2012). Moreover, Xiexin decoction ameliorates blood glucose and decreases basement membrane thickening (Wu et al. 2014). HuoxueJiangtang decoction (ZY) is a traditional Chinese medicine for the treatment of diabetes, and clinical and animal experiments on ZY have confirmed its hypoglycaemic and insulin resistance effects (Zhang, Li, Dong, et al. 2004; Zhang, Li, Zhang, et al. 2004). Our previous studies showed that ZY reduces blood glucose in diabetic rats (Chu et al. 2017). However, it is still unclear whether it has a protective function against kidney damage in diabetic nephropathy rats.

Metformin is an oral hypoglycaemic agent. Evidence suggests that it can alleviate kidney damage in diabetic nephropathy rats (Alhaider et al. 2011). In this study, we constructed a diabetic nephropathy (DN) rat model and evaluated the effects of ZY and metformin + ZY treatment on blood glucose, dietary behaviour, biochemical indices of blood and urine, and renal cortical pathological damage in DN. In addition, we used transcriptome sequencing to find potential genes and signalling pathways for the treatment of DN using ZY.

## Materials and methods

### Animals and drug administration

A total of 70 4-week-old SPF Wistar male rats were obtained from Charles River Laboratories (Beijing, China), and were randomly divided into two groups: 20 rats in the normal group (control) and 50 rats in the diabetic nephropathy model rats (DN) group. The 50 diabetic model rats were established by continuous high-sugar and high-fat feeding, including 67.5% basic feed, 15% lard, 15% sugar, 2% cholesterol (CHOL), and 0.5% bile salt, for 4 weeks, followed by a tail vein injection of 30 mg/kg streptozotocin (30 mg/kg; Sigma-Aldrich, St. Louis, MO, USA). At the same time, the 20 rats in the normal group received normal feed and were injected with an equal amount of saline. After 1 week, all rats were fasted for 12 h and then subjected to fasting blood glucose analysis and the oral glucose tolerance test (OGTT). Before the OGTT, all rats were given a glucose solution by gavage at 3 g/kg, and then blood was taken 1 and 2 h later for the OGTT. The successfully constructed diabetic rats were studied as representative of DN. All animal experiments were conducted following approval from the Research Ethics Committee of Shenzhen Traditional Chinese Medicine Hospital.

The experimental rats were divided into six groups of 10 rats: normal (control), DN, DN + ZY, DN + metformin, DN + metformin + ZY, and DN + metformin + captopril (positive control) groups. Among the groups, 6.25 g/kg ZY, 250 mg/kg metformin (Boyda et al. 2014; Duca et al. 2015; Zhang et al. 2017), and 17.5 mg/kg captopril (Zhang et al. 2017) were given to the rats by gavage once a day for 16 weeks. The details of composition of ZY were shown in Table 1. And the plant ingredients were purchased from Kangmei Pharmaceutical Co., Ltd (Puning, China). All ZY preparations were completed by Shenzhen Key Laboratory of Hospital Chinese Medicine Preparation, Shenzhen Hospital of Traditional Chinese Medicine. The preparation process was as follows: after being soaked in distilled water for 2 h, the medicine was decocted on high fire and continued to be decocted on low fire for 30 min, a total of two times. The obtained medicine juice was mixed, filtered, concentrated into 4 g/mL, and stored in the refrigerator at 4 °C for later use. Metformin (#M107827-100 g, 97% purity) and captopril (#C129108-25 g, 98% purity) were purchased from Aladdin Bio-Chem Technology (Shanghai, China). Another 10 normal rats were treated with ZY (control + ZY), which were used for histopathological analysis to exclude the kidney toxicity of ZY to rats.

**Table 1.** Detailed information on the composition of HuoxueJiangtang decoction (ZY).

Latin binomial	Family	Genus	Herbal name	Batch number	Quantity
*Rehmannia glutinosa* (Gaertn.) DC.	Orobanchaceae	Rehmannia	Rehmanniae Radix	181200019	20 g
*Astragalus membranaceus* Moench	Fabaceae	Astragalus	Astragali Radix	181200171	30 g
*Ophiopogon japonicus* (Linn. f.) Ker-Gawl.	Asparagaceae	Ophiopogon	Ophiopogonis Radix	181105081	15 g
*Pseudostellaria heterophylla* (Miq.) Pax	Caryophyllaceae	Pseudostellaria	Pseudostellariae Radix	181000361	30 g
*Carthamus tinctorius* Linn.	Asteraceae	Carthamus	Carthamus tinctorious	181102711	12 g
*Dioscorea japonica* Thunb.	Dioscoreaceae	Dioscorea	Rhizoma Dioscoreae	1901101251	12 g
*Prunus persica* (L.) Batsch	Rosaceae	Prunus	Semen Persicae	181001851	10 g
*Rheum officinale* Baill.	Polygonaceae	Rheum	Radix et Rhizoma Rhei	181000099	16 g
*Schisandra chinensis* (Turcz.) Baill.	Schisandraceae	Schisandra	Schisandrae Chinensis Fructus	1801023	15 g
*Polygonatum kingianum* Coll. et Hemsl.	Asparagaceae	Polygonatum	Polygonati Rhizoma	171202	15 g
*Salvia miltiorrhiza* Bunge	Lamiaceae	Salvia	Salviae Miltiorrhizae Radix et Rhizoma	18040901	30 g
*Paeonia suffruticosa* Andr.	Paeoniaceae	Paeonia	Moutan Cortex	180203261	12 g

### Biochemical analysis

After 16 weeks of administration, the water intake and food intake of the rats in each group were recorded. Blood was collected and centrifuged at 3000 *g* for 20 min using an automatic biochemical analyzer (Cobas 8000 c702; Roche Diagnostics, Mannheim, Germany). The serum triglyceride (TG), serum CHOL, serum low-density-lipoprotein CHOL (LDL-CH), serum high-density-lipoprotein CHOL (HDL-CH), serum urea, and serum creatinine (SCr) levels were measured in each group of rats. In addition, 24 h after the last drug treatment, the total urine volume from each rat was recorded using a rat metabolic cage, and the urine total protein (UTP) and urine albumin content were also determined by a biochemical analyzer.

### Histopathological analysis

After 16 weeks of administration, the renal cortex of rats in each group was collected and prepared for transmission electron microscopy (Philips CM 200). The changes in the glomerular ultrastructure of rats in each group were observed. The glomerular basement membrane (GBM) was analyzed using high-resolution images obtained by transmission electron microscopy (Basta-Jovanovic et al. 1990). Another part of the renal cortex was fixed in paraffin-embedded sections by 10% formalin for 24 h followed by haematoxylin and eosin (H&E) staining, periodic acid–Schiff (PAS) staining, and Masson’s trichrome staining, in accordance with the kit manufacturer’s instructions. All images for histopathological analysis were taken by light microscopy at 200× and 800× magnification.

### RNA sequencing

The three samples of each group were used for the RNA sequencing analysis. Total RNA was isolated using Trizol reagent (Invitrogen, Carlsbad, CA, USA), in accordance with the standard protocol. Before RNA library construction, rRNA was removed using the RiboMinus Eukaryote Kit (Qiagen, Hilden, Germany). For RNA library preparation, the NEB Next Ultra Directional RNA Library Prep Kit for Illumina (NEB, Ipswich, MA, USA) was used following the standard manufacturer’s recommendations. Then, libraries were run on an Illumina HiSeq 2000 platform for sequencing.

Raw data sequencing by the Illumina HiSeq 2000 platform was quantified, and low-quality data were removed. After filtration and mapping to human reference genome hg19 using TopHat with the default parameters, clean reads were obtained. These reads were used for subsequent analysis. The gene expression level was normalized using the reads per kilobase of exon model per million mapped reads (RPKM) method. Differentially expressed genes were defined as those with a fold difference of more than 1.5-fold.

### Bioinformatics

Heatmaps and the Kyoto Encyclopaedia of Genes and Genomes (KEGG) pathways were drawn and enriched using the R package. The KEGG pathways that met the criteria of false discovery rates ≤0.001 were considered to be significant.

### Statistics

All data were analysed using SPSS version 18.0 (SPSS Inc., IL, USA). Data are presented as mean ± standard deviation. Comparisons of observations between groups were performed using one-way analysis of variance. *p* < 0.05 was considered to be statistically significant.

## Results

### Effects of ZY on blood glucose, food intake, water intake, and biochemical parameters of DN

**Figure 1.**
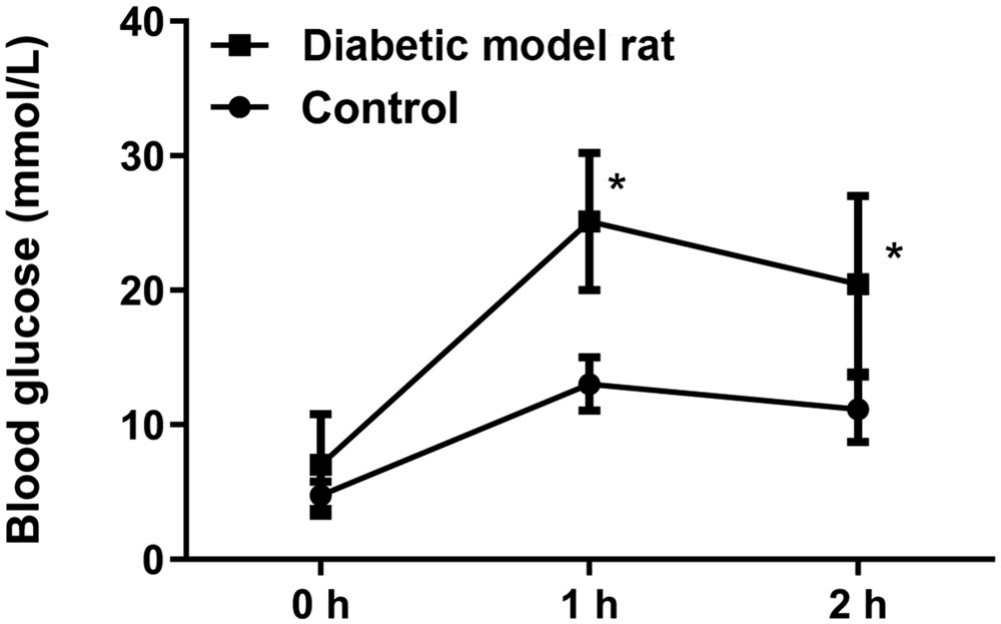
OGTT showed blood glucose levels in diabetic model rats and normal rats (control). OGTT: oral glucose tolerance test; *p < 0.05 vs. control.

We first constructed a diabetic rat model by high-sugar and high-fat feeding. The OGTT results showed that the blood glucose level in the diabetic model rat group was higher than that in the control group at 1 and 2 h after the intragastric administration of 3 g/kg glucose solution. The blood glucose level rose to a peak at 1 h, and was still close to this blood glucose level at 2 h in the diabetic model rat group, suggesting that the diabetic rat model had been successfully constructed (Figure 1). The successfully constructed diabetic model rats were studied as a model of DN.

**Figure 2.**
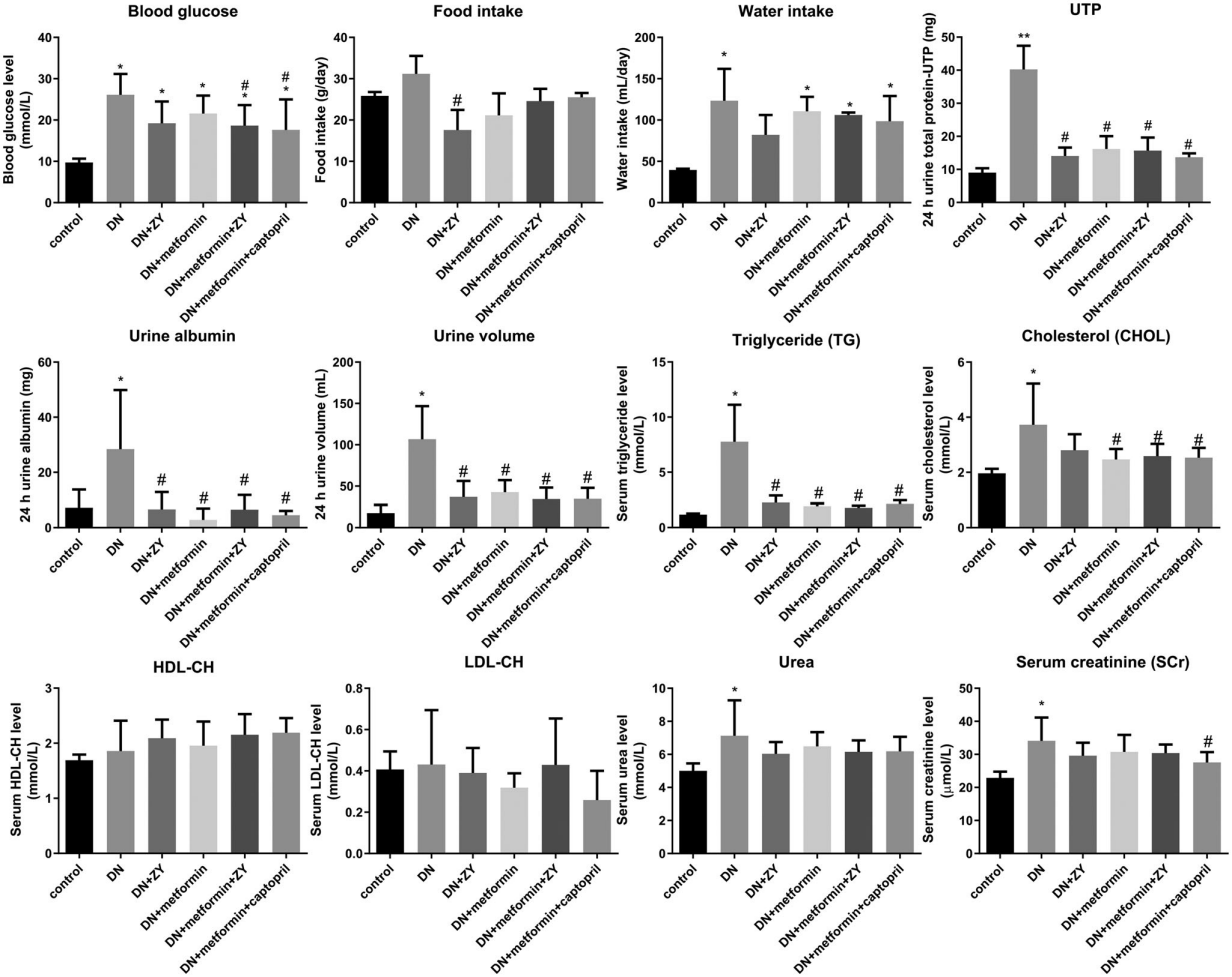
Effects of ZY on blood glucose, food intake, water intake, and biochemical parameters of DN. (A) Blood glucose, (B) food intake, (C) water intake, (D) 24 h urine total protein, (E) urine albumin, (F) urine volume, (G) serum triglyceride, (H) serum cholesterol, (I) serum HDL-CH, (J) serum LDL-CH, (K) serum urea, and (L) serum creatinine levels in the normal rats (control) and in DN with different treatments. ZY: HuoxueJiangtang decoction; DN: diabetic nephropathy model rat; HDL-CH: high-density lipoprotein-CHOL; LDL-CH: low-density lipoprotein-CHOL; DN + ZY: DN treated with ZY; DN + metformin: DN treated with metformin; DN + metformin + ZY: DN treated with metformin combined with ZY; DN + metformin + captopril: DN treated with metformin combined with captopril. *p < 0.05 vs. control; ^#^*p* < 0.05 vs. DN.

To test the effect of ZY alone or metformin + ZY on DN, blood glucose, food intake, water intake, and blood and urine indicators were evaluated. The results showed that, compared with the levels in the control group, the blood glucose (26.11 mmol/L), daily food intake (31.18 g), and daily water intake (123.47 mL) levels in the DN group were increased (Figure 2(A–C)). Compared with the levels in the DN group, ZY, metformin, metformin + ZY, and metformin + captopril were able to lower blood glucose, food intake, and water intake (Figure 2(A–C)). Compared with the levels in the ZY and metformin groups, the blood sugar levels in the metformin + ZY and metformin + captopril groups also decreased, indicating that ZY combined with metformin treatment exerted a better effect of lowering blood sugar (Figure 2(C)). Differences in urine parameters between the groups were also observed. Compared with those in the control group, the levels of urine volume (69.90 mL), urine total protein (40.23 mg), and urine albumin (28.41 mg) at 24 h in the DN group were significantly increased; however, these levels in the ZY, metformin, metformin + ZY, and metformin + captopril groups were significantly lower than those in the DN group (Figure 2(D–F)).

Data on TG, CHOL, HDL-CH, LDL-CH, urea, and creatinine in serum showed that the TG (8.27 mmol/L) content in the DN group was significantly increased; however, the TG content in the drug-treated group was significantly lower than that in the DN group, and there was no significant difference between the groups treated with different drugs (Figure 2(G)). The serum CHOL (3.73 mmol/L) level in the DN group was significantly increased. ZY treatment did not affect the CHOL level in the DN group; however, other drug treatments significantly reduced the serum CHOL content in the DN group (Figure 2(H)). As shown in Figure 2(I–J), the HDL-CH and LDL-CH contents in serum showed no significant changes among the drug-treated groups. As shown in Figure 2(K–L), the increases of urea and creatinine levels in the DN group were recovered in rats treated with different drugs.

### Pathological changes of DN treated with ZY

**Figure 3.**
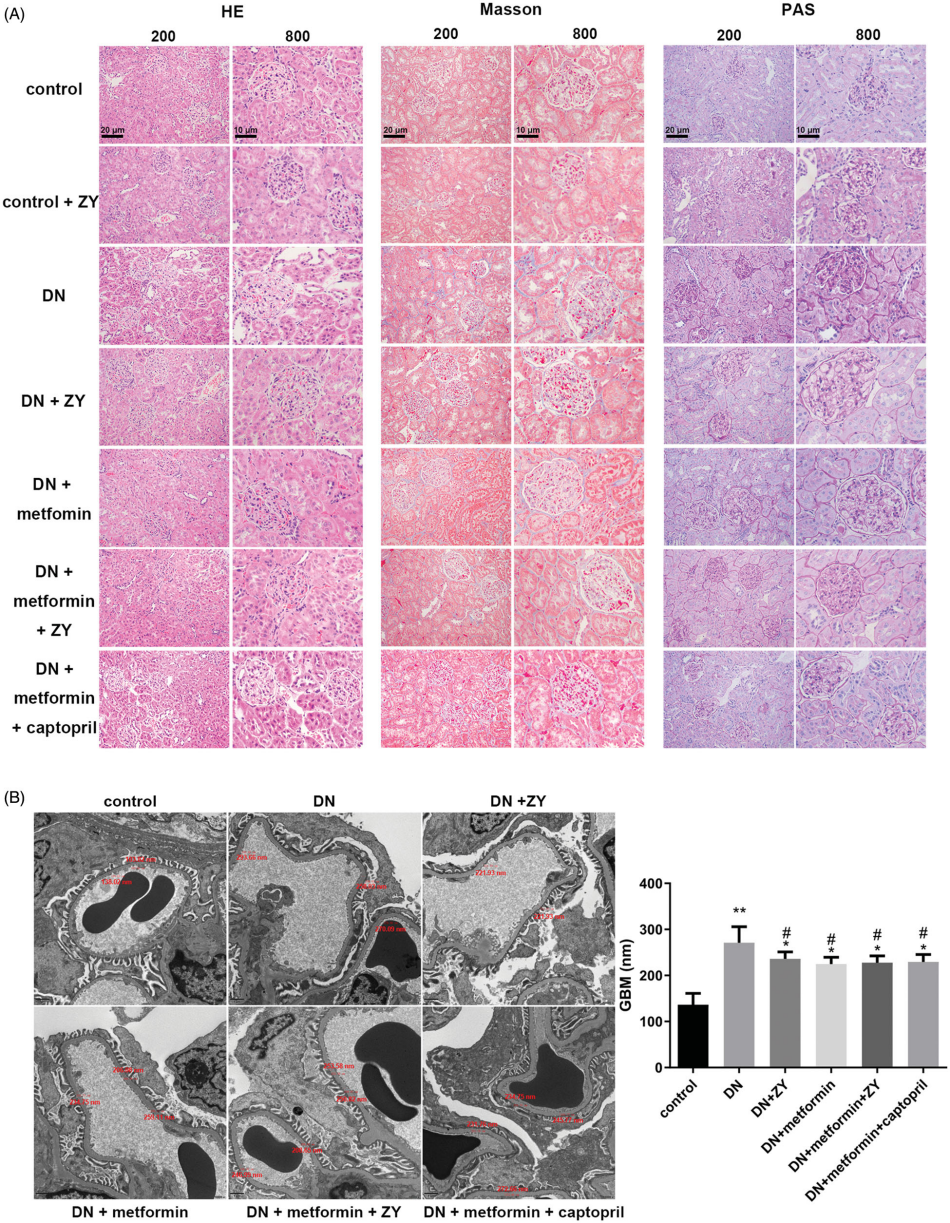
Histopathological analysis showed changes in the renal cortex in response to different drug treatments. (A) H&E staining, PAS staining, and Masson’s trichrome staining showed changes in the normal rats (control) and model rats with different drug treatments. (B) Transmission electron microscopy showed the effects of different drug treatments on the glomerular ultrastructure. GBMs were quantified based on these representative images. H&E: haematoxylin and eosin; PAS: Periodic Acid–Schiff; GBMs: glomerular basement membranes; ZY: HuoxueJiangtang decoction; DN: diabetic nephropathy model rat; control + ZY: normal rats treated with ZY; DN + ZY: DN treated with ZY; DN + metformin: DN treated with metformin; DN + metformin + ZY: DN treated with metformin combined with ZY; DN + metformin + captopril: DN treated with metformin combined with captopril. Bars represent 10 and 20 μm. *p < 0.05 vs. control; **p < 0.01 vs. control; ^#^*p* < 0.05 vs. DN.

To understand the effect of ZY alone or metformin + ZY on DN, pathological staining of the renal cortex was performed in each group, and electron microscopy was used to observe the structural change in glomeruli. The H&E staining results showed that the renal tissue structure was tightly arranged, and the glomerular and tubular structures were normal in the control and control + ZY groups (Figure 3(A)). The renal lesions were obvious, the GBM was thickened, and the extracellular matrix and mesangial matrix were accumulated in the model groups (Figure 3(A)). The pathological changes were improved in the drug-administered groups, compared with those in the DN groups (Figure 3(A)). Masson’s trichrome staining results showed that there was no obvious blue staining in the kidney tissue in the control and control + ZY groups, suggesting that there was a very small number of collagen fibres (Figure 3(A)). The number of collagen fibres was increased in the DN group, compared with that in the control group, indicating that diabetic nephropathy promoted renal fibrosis (Figure 3(A)). The collagen fibres in the kidney tissue in each drug-administered group were at lower levels than those in the DN group (Figure 3(A)). There was no difference in the expression of collagen fibres in the kidney tissue between the ZY group and the metformin group (Figure 3(A)). The expression levels of collagen fibres in the kidney tissue of the metformin + ZY and metformin + captopril groups were lower, being close to that of the control group (Figure 3(A)). The PAS staining results showed that the polysaccharide and glycogen expression levels in the renal tissue of the DN group were significantly higher than those of the control and control + ZY groups (Figure 3(A)). The expression levels of polysaccharide and glycogen in the renal tissue of each drug-administered group were lower than those in the DN group (Figure 3(A)). The expression levels of polysaccharide and glycogen in the kidney tissue of the ZY and metformin groups were similar, whereas those in the metformin + ZY and metformin + captopril groups were lower (Figure 3(A)). To sum up, the findings showed that DN had been successfully established. ZY or metformin alone could improve the pathological changes of kidney tissue, and therapy involving ZY combined with metformin showed a better effect.

Electron microscopy showed that the average thickness of GBM in the kidney tissue of the control group was 139.97 nm, and the podocytes were arranged neatly. The GBM of the DN group was evenly thickened, and its maximum thickness was 293.66 nm, with an average of 272.34 nm. In addition, the renal podocytes merged without dense deposition in the DN group. Compared with that in the DN group, the GBM thickness of rats in the different drug administration groups was uniformly reduced, and the degree of podocyte fusion was significantly reduced (Figure 3(B)).

### Effect of ZY on gene expression of the renal cortex in DN

**Figure 4.**
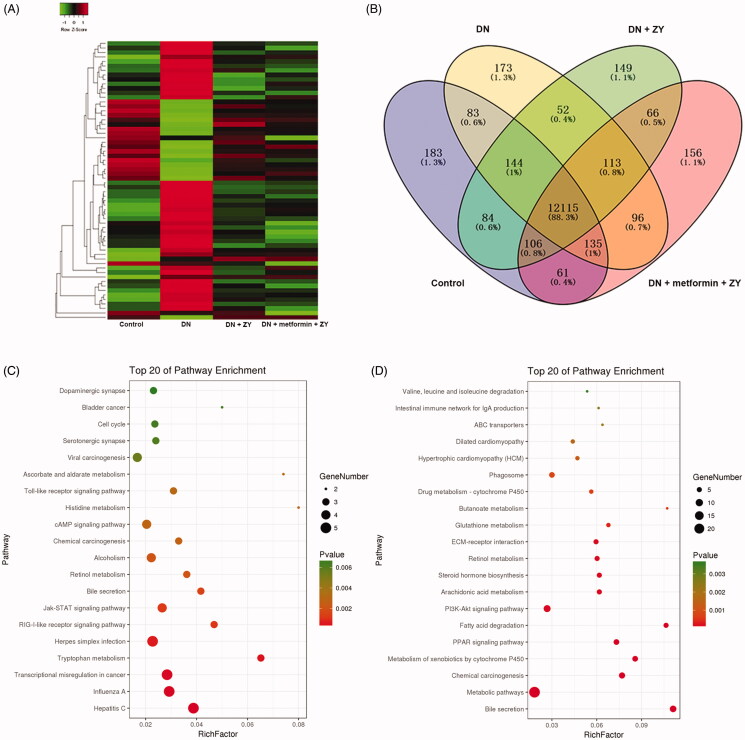
RNA sequencing indicated differentially expressed genes in DN and DN treated with different drugs. (A) The heatmap showed the differentially expressed genes. (B) The Venn diagram showed commonly and specifically expressed genes in each group of rats. (C) KEGG enrichment analysis showed the top 20 most differentially expressed genes in the control group compared with the DN group. (D) KEGG enrichment analysis showed the top 20 most differentially expressed genes in the DN + metformin group compared with the DN + metformin + ZY group. ZY: HuoxueJiangtang decoction; DN: diabetic nephropathy model rat; KEGG: Kyoto Encyclopaedia of Genes and Genomes; DN + ZY: DN treated with ZY; DN + metformin: DN treated with metformin; DN + metformin + ZY: DN treated with metformin combined with ZY; DN + metformin + captopril: DN treated with metformin combined with captopril.

**Figure 5.**
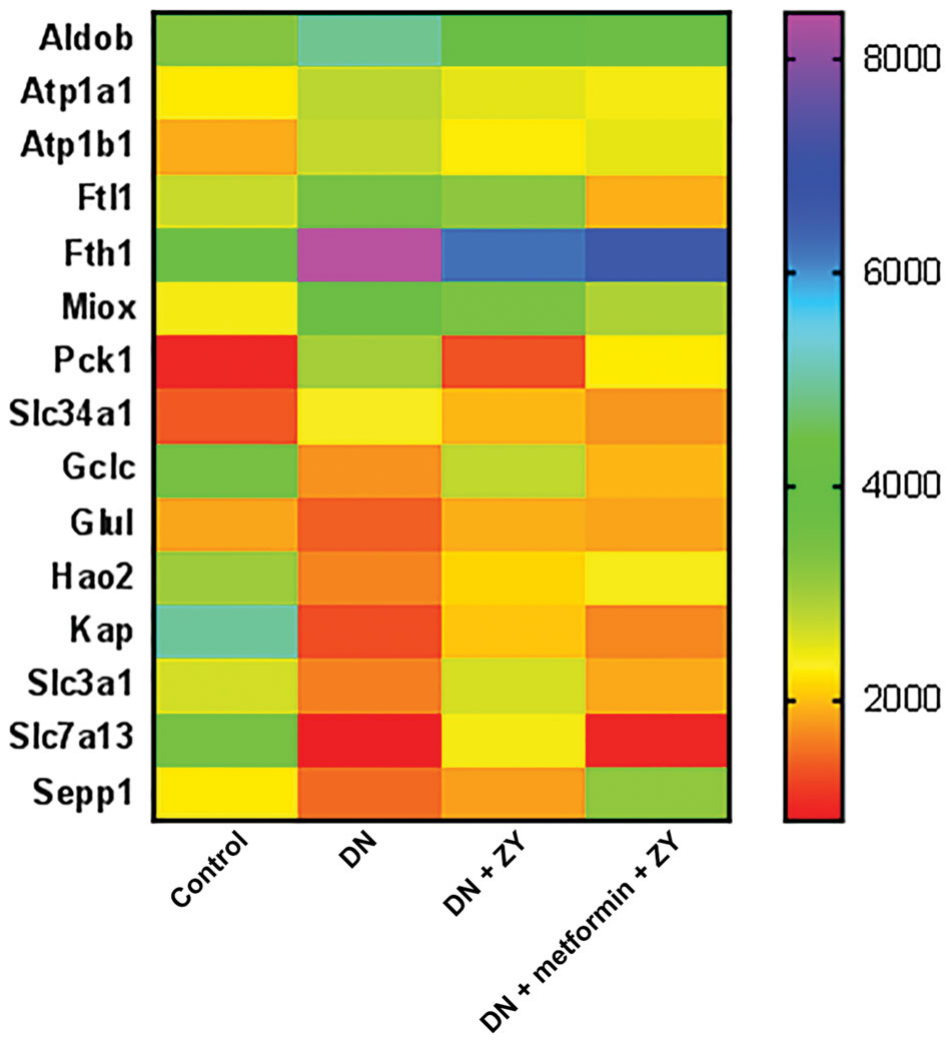
The heatmap generated with RNA sequencing data showed the differentially expressed genes in DN treated with different drugs. ZY: HuoxueJiangtang decoction; DN: diabetic nephropathy model rat; DN + ZY: DN treated with ZY; DN + metformin + ZY: DN treated with metformin combined with ZY.

To study the molecular mechanism of ZY protection against renal tissue damage, we performed RNA sequencing. The RNA sequencing results showed that there were 12,115 genes co-expressed in the control, DN, DN + ZY, and DN + metformin + ZY groups, accounting for 88.3% of the differentially expressed genes (Figure 4(A,B)). The KEGG pathway enrichment results showed that the genes that were differentially expressed in the control vs. DN and DN + metformin vs. DN + metformin + ZY had the same signalling pathways in the top 20 most enriched pathways, including dopaminergic synapse, serotonergic synapse, and cyclic adenosine monophosphate (cAMP) signalling pathway (Figure 4(C,D)). Furthermore, among these differentially expressed genes, 15 genes that were differentially expressed in DN recovered in the ZY alone or metformin + ZY treatment group or their levels were partially restored to the control group level, which were randomly displayed using a heatmap (Figure 5).

## Discussion and conclusion

In the present study, we found that ZY and metformin + ZY treatments achieved hypoglycaemic effects, improved urinary protein levels and serum TG effects. In addition, metformin + ZY treatment significantly inhibited pathological changes of the renal cortex. Transcriptome sequencing analysis indicated that a range of genes were differentially expressed in the DN group, and some genes returned to normal levels in the group with DN treated with different drugs. KEGG pathway enrichment analysis indicated that differentially expressed genes were particularly associated with dopaminergic synapse, serotonergic synapse, and cAMP signalling pathways.

Drinking more, polyuria, and eating more are clinical manifestations of diabetics, and lipoprotein metabolism occurs frequently in people with diabetes (American Diabetes Association [ADA] 2014). In some people with diabetes, abnormal LDL appears, LDL and TG increase, and HDL and CHOL decrease (Howard et al. 1984). In the early stage of diabetes, hyperglycaemia exceeds the reabsorption capacity of the kidney, which can lead to an increase in glomerular filtration, thereby increasing the amount of urine (Kanwar et al. 2008). Proteinuria further aggravates the basement membrane thickening of the glomerulus, mesangial expansion, and glomerular sclerosis. Therefore, the progression of diabetes to nephropathy often manifests as albuminuria, renal dysfunction, and an increased risk of cardiovascular disease (Kitada et al. 2014). Serum urea is one of the diagnostic indicators of kidney injury. It is synthesized in the liver, enters the bloodstream, and is metabolized by the kidney. High serum urea predicts impaired renal function; diabetics have significantly higher serum urea levels than non-diabetics (Prasad et al. 2017). SCr is one of the most widely used indicators for the clinical evaluation of renal function and can estimate the glomerular filtration rate (Inker et al. 2012); moreover, elevated SCr suggests impaired renal function. In our study, blood glucose, urine volume, TG, CHOL, urea, and SCr were significantly increased in DN; however, there was no significant change in blood lipid-related indicators. This may have been caused by the animal models not fully simulating clinical manifestations of the human body (Kitada et al. 2016). We also observed that ZY alone and metformin + ZY treatments inhibited kidney damage in DN. Hyperglycaemia, urinary protein, GBM thickening, and other phenotypes were reversed. In particular, metformin + ZY treatment also inhibited the level of CHOL in DN, suggesting that metformin + ZY treatment has a protective effect on blood glucose and metabolic function in DN.

Renal histopathological changes in diabetic nephropathy include mesangial matrix deposition, GBM thickening, and tubular atrophy, which eventually cause interstitial fibrosis and glomerular sclerosis (Schwartz et al. 1998; Umanath and Lewis 2018). Among these, GBM thickening is the most easily recognised histological feature of diabetic nephropathy, and it is the easiest to quantify in diabetic nephropathy patients and animal models (Alpers and Hudkins 2018). In our study, we used H&E staining, Masson’s trichrome staining, and PAS staining to observe DN tissue lesions. We confirmed pathological changes such as renal fibrosis and GBM thickening in DN, and metformin + ZY improved these tissue changes.

Accompanied by improvements of blood glucose, blood lipid, metabolism, and renal morphological conditions in DN, genes of the ZY- and metformin + ZY-treated rats also showed recovery of expression. It is worth noting that the differentially expressed genes, when compared with the levels in the DN group, were particularly associated with dopaminergic synapse, serotonergic synapse, and cAMP signalling pathways. These commonly differentially expressed genes and pathways might be involved in the regulation of the pathogenic mechanisms of DN as well as the therapeutic response of ZY alone or metformin + ZY in the protection of blood glucose, metabolism, and renal injury in DN. Studies have shown that both spontaneous type 1 diabetes and type 2 diabetes model rats show neurological synaptic degeneration and neuronal decline (Nakano et al. 2016; Haider et al. 2019). The cAMP pathway also plays a regulatory role in multiple tissues, including in diabetes, and is closely related to the renal fibrosis of diabetic nephropathy (Bockus and Humphries 2015; Arcaro et al. 2018; Huang et al. 2018). The above evidence shows that dopaminergic synapse, serotonergic synapse, and cAMP pathway might be related to the mitigating effect of metformin + ZY on DN.

Here, we used a heatmap to randomly display 15 genes that were differentially expressed in DN. We found that there was no significant difference in the ZY or metformin + ZY treatment group, compared with the control group. Among these genes, that encoding myoinositol oxygenase (Miox), a tubular enzyme, was reported to be activated in a high-fat-diet-induced diabetic animal model and to promote diabetic tubular damage (Tominaga et al. 2016). Phosphoenolpyruvate carboxykinase 1 (Pck1) is a key gluconeogenic enzyme found to maintain glucose homeostasis in diabetic animals (Hu et al. 2019). In the present study, we found that Miox and Pck1 were downregulated after metformin + ZY treatment, suggesting that they may be involved in the protective effect of metformin + ZY against DN. The specific adjustment function for Miox and Pck1 genes will be further studied.

Taking the findings together, our study found that metformin + ZY treatment had a beneficial effect on DN. We proposed that the alleviation of Pck1 and Miox genes, and dopaminergic synapse, serotonergic synapse, and cAMP signalling pathways were associated with the role of metformin + ZY treatment. However, there are some limitations in our study. Our study is a preliminary study of the basic theoretical mechanism. We regret that we are unable to provide the LC-MS chemical fingerprint for each plant ingredient and the final decoction. In the future, the appropriate chemical fingerprint (TLC, HPLC, GC, MS, or NMR) of the herbal prescription will be performed to explore the specific mechanism of TCM. And we will verify the sequencing results in cell experiments in the future to further explore the underlying mechanism.

## Data Availability

The authors declare that the data are available.
